# Comparison of nested case-control and survival analysis methodologies for analysis of time-dependent exposure

**DOI:** 10.1186/1471-2288-5-5

**Published:** 2005-01-25

**Authors:** Vidal Essebag, Robert W Platt, Michal Abrahamowicz, Louise Pilote

**Affiliations:** 1 Division of Cardiology, Beth Israel Deaconess Medical Center, Harvard University, Boston, MA, USA; 2 Department of Epidemiology and Biostatistics, McGill University, Montreal, Canada; 3 Division of Clinical Epidemiology, McGill University Health Center, Montreal, Canada

## Abstract

**Background:**

Epidemiological studies of exposures that vary with time require an additional level of methodological complexity to account for the time-dependence of exposure. This study compares a nested case-control approach for the study of time-dependent exposure with cohort analysis using Cox regression including time-dependent covariates.

**Methods:**

A cohort of 1340 subjects with four fixed and seven time-dependent covariates was used for this study. Nested case-control analyses were repeated 100 times for each of 4, 8, 16, 32, and 64 controls per case, and point estimates were compared to those obtained using Cox regression on the full cohort. Computational efficiencies were evaluated by comparing central processing unit times required for analysis of the cohort at sizes 1, 2, 4, 8, 16, and 32 times its initial size.

**Results:**

Nested case-control analyses yielded results that were similar to results of Cox regression on the full cohort. Cox regression was found to be 125 times slower than the nested case-control approach (using four controls per case).

**Conclusions:**

The nested case-control approach is a useful alternative for cohort analysis when studying time-dependent exposures. Its superior computational efficiency may be particularly useful when studying rare outcomes in databases, where the ability to analyze larger sample sizes can improve the power of the study.

## Background

The nested case-control design employs a case-control approach within an established cohort [1,2] to obtain estimates from a sample of the cohort that are similar to estimates obtained from analysis of the entire cohort [3,4]. The nested case-control design is being increasingly used in large cohorts of patients from prospective studies and randomized clinical trials. This design has become popular because it allows for statistically efficient analysis of data from a cohort with substantial savings in cost and time [5,6].

When studying exposures that vary with time, an additional level of complexity is introduced by the need to account for time-dependent exposure in both the design and analysis [7,8]. This can be accomplished by including time-dependent covariates in a Cox proportional-hazards regression model [9]. Alternatively, a nested case-control approach can be used provided that the exposure and covariate information for controls reflects values corresponding to the time of selection of their respective case.

This study compares nested case-control and survival analysis methodologies for evaluating time-dependent exposure. The risk of pacemaker insertion associated with dosage of amiodarone (an anti-arrhythmic medication used for the treatment of atrial fibrillation (AF)), represented by a time-dependent covariate, is evaluated in cohort of patients with AF using both methods for illustrative purposes. The comparability of results is evaluated and differences in computational efficiency are quantified for increasing cohort sizes. Advantages and limitations of the respective methodologies are discussed.

## Methods

### Study cohort

A cohort of 11395 elderly (>65 years of age) Quebec residents with AF and a myocardial infarction (MI) between 1991 and 2000 was created by linking the provincial hospital discharge summary database with the provincial physician and drug claims database, using methods described previously [10]. Approval for the study was obtained from the McGill University Faculty of Medicine Institutional Review Board.

In order to evaluate the effect of amiodarone dose on the previously demonstrated association between amiodarone therapy for AF and an increased risk of permanent pacemaker requirement [10], only patients newly started on amiodarone after their diagnosis of AF were included in the study cohort. Amiodarone dose was represented as a binary time-dependent variable comparing daily doses >200 mg to ≤200 mg. Covariate information included age, sex, calendar year of cohort entry, baseline sinus node or conduction disorder, ventricular arrhythmia, and time-dependent exposure to five categories of medications. The final study cohort included 1340 subjects followed from the date of their first prescription of amiodarone until the first of pacemaker implantation, death, or March 31, 2001.

### Statistical analysis

The data for the entire cohort of 1340 subjects including all fixed and time-dependent variables was represented in counting process notation suitable for Cox regression of time-dependent exposure [11]. Multiple records (with consecutive start and end times) were created for each subject to account for every change in exposure to any of the time-dependent variables over the study period.

The hazard ratio (HR) of pacemaker insertion associated with amiodarone doses >200 mg per day was estimated using a Cox proportional-hazards model including all fixed and time-dependent covariates. The timescale used in the model was time since first prescription of amiodarone. Non-significant variables (other than age and sex) were sequentially removed if the resultant model had no significant increase in Akaike Information Criteria (AIC) and no significant change in the HR for amiodarone dose.

The nested case-control approach was also used to estimate the HR of pacemaker insertion associated with amiodarone doses >200 mg per day. Cases of pacemaker insertion were identified and controls were randomly selected from the risk-set of each case (i.e. subjects present in the cohort at the time the case is defined). After selecting all controls and recording their index dates (i.e. the time, in cohort time, at which the respective case is defined) the relevant time-dependent covariate information was retrieved by merging with the database configured in counting process notation. The relevant subject record was selected by requiring that the index date fall within the start and end time of the subject record for each control.

The nested case-control approach was repeated using 4, 8, 16, 32, and 64 controls per case. For each number of controls per case, random sampling of controls for all cases and conditional logistic regression analysis was repeated 100 times using the OUTEST option in the PROC PHREG statement to create an output SAS data containing all the parameter estimates [11]. The mean and standard deviation (SD) of the parameter estimates for each number of controls per case was calculated.

Computational times for regression models of time-dependent exposures using nested case-control and survival analysis methodologies were compared. The nested case-control samples with 4 and 32 controls per case were analyzed using conditional logistic regression with the PHREG procedure in SAS Release 8.2 [12]. The full cohort was analyzed using Cox regression adapted for analysis of time-dependent covariates with the PHREG procedure in SAS Release 8.2. Ties were handled using the TIES = EFRON option in the PHREG procedure [11]. All analyses were performed using an Intel Pentium 4 computer with a 1.80 GHz central processing unit (CPU) and 256 MB of random access memory (RAM).

Relative computational efficiencies were evaluated by comparing the CPU times of the three regression models used to analyze the cohort. Relative increases in computational time as a function of sample size were quantified by repeating the analyses on progressively larger cohorts. This was done by progressive doubling of the original cohort to 2, 4, 8, and 32 times its original size. Given that the objective was to compare computational times, all fixed and time-dependent exposures were included in all models regardless of statistical significance.

The computational efficiency of the Cox regression model was also compared to the conditional logistic regression model where the nested case-control sample included all possible controls for each case. This comparison was performed for the original cohort of 1340 subjects with 53 cases (including 2 ties). All analyses were performed using the PHREG procedure. Ties were handled using the TIES = EFRON option in the PHREG procedure, and subsequently using the TIES = DISCRETE option in the PHREG procedure for comparison.

## Results

### Comparative risk estimates

Pacemaker implantation occurred in 53 of the 1340 subjects during the study period. In the final Cox regression model, amiodarone daily dose (>200 mg vs. ≤200 mg) was associated with an increased risk of pacemaker insertion (HR: 2.03; 95 percent confidence interval: 1.00, 4.14; *p *= 0.05; Parameter Estimate: 0.71; Standard Error of Parameter Estimate: 0.36), after adjusting for age, sex, as well as baseline sinus node or conduction disorder (the only covariate that was an independent predictor of outcome).

The results of 500 nested case-control analyses (i.e. 100 for each of 4, 8, 16, 32, and 64 controls per case) are summarized in Table 1. When using any number from 4 to 64 controls per case, the mean point estimate (of 100 analyses repeating the random sampling of controls) of the parameter estimate (and HR) was very similar to that obtained using Cox regression on the full cohort (i.e. HR: 2.03; Parameter Estimate: 0.71). The SDs of the parameter estimates decreased with increasing numbers of controls per case (Table 1).

**Table 1:** Nested case-control analyses with repeated sampling for increasing numbers of controls per case: Hazard ratio of pacemaker insertion associated with amiodarone dose* in 1340 elderly Quebec residents with atrial fibrillation

Controls per Case (n)	Repeated Sampling (n)	Mean HR†	SD† of HR	Min HR	Max HR	Mean Parameter Estimate	SD of Parameter Estimates
4	100	2.14	0.51	1.27	3.59	0.73	0.23
8	100	2.19	0.41	1.39	3.86	0.77	0.18
16	100	2.02	0.22	1.55	2.51	0.70	0.11
32	100	2.07	0.15	1.78	2.55	0.73	0.07
64	100	2.02	0.11	1.82	2.33	0.70	0.05

* The effect of amiodarone dose, represented by a binary time-dependent covariate (>200 mg vs. ≤200 mg), was adjusted for age, sex, and baseline sinus node or conduction disorder in all models.

† HR, hazard ratio; SD, standard deviation.

### Comparative computational efficiencies

**Figure 1:**
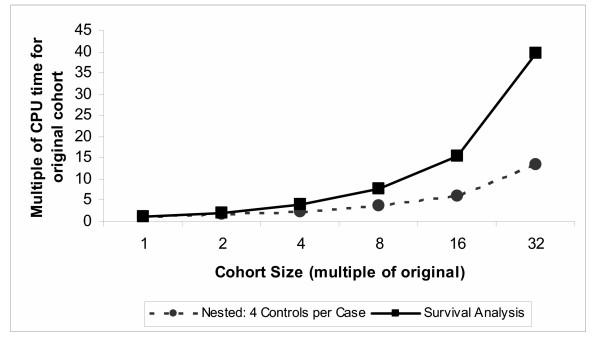
Increase in computational time with increasing sample size for nested case-control and survival analysis of cohort data with time-dependent covariates

The computational time required to analyze the cohort of 1340 subjects with 53 events (cases) in models including four fixed variables and seven time-dependent variables is presented in Table 2. CPU times are displayed for the Cox regression models and the nested case-control regression models (with 4 or 32 controls per case). For the cohort in its initial size, using 32 rather than 4 controls per case increased CPU time by a factor of 3, whereas using Cox regression increased CPU time by a factor of 42. As the cohort size was increased to 32 times the original (i.e. 42880 subjects), the increase in CPU time was greater for the Cox regression model than the nested case-control models. Figure 1 displays graphically the increase in CPU time with increasing sample size for nested case-control and Cox regression models. The relative computational efficiency was magnified as the sample size increased, such that the CPU time for Cox regression with a cohort of 42880 subjects was 125 times greater than the nested case-control model with 4 controls per case (Table 2).

**Table 2:** Computational times for nested case-control and survival analyses of time-dependent data for cohorts of increasing sizes: Models* of the risk of pacemaker insertion in elderly Quebec residents with atrial fibrillation

Cohort Size†: #Subjects (#Cases)	1340 (53)	2680 (106)	5360 (212)	10720 (424)	21440 (848)	42880 (1696)
Cohort Size: multiple of original	1	2	4	8	16	32
CPU‡ Time (seconds):						
Nested: 4 Controls per Case	0.03	0.05	0.07	0.11	0.18	0.4
Nested: 32 Controls per Case	0.08	0.1	0.2	0.41	0.7	1.49
Survival Analysis	1.26	2.51	5.06	9.54	19.53	49.91
CPU Time (multiple of Nested 4):						
Nested: 4 Controls per Case	1	1	1	1	1	1
Nested: 32 Controls per Case	3	2	3	4	4	4
Survival Analysis	42	50	72	87	109	125

* All models included four fixed variables (age, sex, calendar year of cohort entry, and baseline sinus node or conduction disorder) and seven time-dependent variables (amiodarone dose, ventricular arrhythmia, and exposure to sotalol, class I antiarrhythmic agents, beta-blockers, calcium channel blockers, and digoxin).

† The original cohort of 1340 subjects was increased by progressive duplication to 2, 4, 8, 16, and 32 times its original size.

‡ CPU, central processing unit.

The computational time of the Cox regression model was also compared to the nested case-control model including all possible controls for each case. When ties were handled using the TIES = EFRON option, the CPU time for Cox regression was 1.06 times greater than the CPU time for nested case-control model (1.26 vs. 1.19 seconds). When ties were handled using the TIES = DISCRETE option, the CPU time for Cox regression was 3.91 times greater than the CPU time for nested case-control model (14.65 vs. 3.75 seconds).

## Discussion

In this study we illustrate empirically that a nested case-control approach can be used to analyze a cohort with time-dependent covariates, with results that are similar to those obtained by Cox regression. Additionally, given that the nested case-control approach obviates the computationally intensive calculations involved in Cox regression when time-dependent covariates are used, the example also illustrates quantitatively the large reduction in CPU time required for analysis.

The similarity between the two methodologies is expected given that conditional logistic regression used to analyze nested case-control studies (as well as other matched case-control studies) is based on inference procedures adapted from Cox regression; i.e. the conditional likelihood used in conditional logistic regression is exactly the same form as the partial likelihood used in Cox regression except that the denominator includes only a selected number of sampled controls as opposed to all subjects available in the risk set [13]. The inclusion of time-dependent covariates adds an additional level of complexity to the analysis but remains based on the same inference procedures.

The statistical efficiency of the nested case-control approach for cohort analysis depends on the number of controls per case selected. Our example demonstrates the expected decrease in the SD of the parameter estimates as the number of controls per case increases. This decrease in variance is explained by the fact that as the number of controls per case increases (towards the total number of controls in a case's risk-set), the probability of choosing the same controls increases, as does the proportion of available controls selected (i.e. approximating the situation in Cox regression where every case is compared to all controls in its risk-set).

In general, the use of 4 controls per case provides a relative statistical efficiency of 0.8 compared to the use of an infinite number of controls [14]. However, the relative efficiency also depends on the probability of exposure among the controls and on the magnitude of the estimated relative risk. Gains in statistical efficiency are possible by using greater than 4 controls per case particularly when the probability of exposure among the controls is <0.1 [4,15]. In addition to situations where exposure prevalence in controls is low, increasing the number of controls per case is beneficial when the number of case-control sets is small [16].

The major reason for the superior computational efficiency of the conditional logistic regression method for nested-case control analysis of time-dependent covariates is that only a sample of all possible controls are included in the risk set of each case (whereas all are included in Cox regression). As illustrated in Figure 1 and Table 2, the impact on computational efficiency of sampling a fixed number of controls per case is greater for larger cohorts because the sample of controls represents a smaller proportion of the all the possible controls for each case. While this effect of sampling controls may be the main reason for the computational efficiency of the nested case-control approach is not the only reason. As demonstrated, even when all possible controls are included in the risk set of each case, the computational time of the conditional logistic regression increases significantly but remains faster than Cox regression. This is because the two analyses process time-dependent covariates differently. In Cox regression, risk sets and time-dependent covariates are calculated at the time of each case failure. In conditional logistic regression, the risk sets and time-dependent covariates are calculated in advance. The relative efficiency also depends on how ties are handled, with Cox regression relatively less efficient when ties are handled using the TIES = DISCRETE option compared to the TIES = EFRON option.

The nested case-control approach for cohort analysis offers some advantages over analysis of an entire cohort that may be important regardless of the type of cohort used. A potential advantage with respect to design is the option to match controls to cases on the basis of possible confounding covariates for which estimation of effect is not of interest. Another advantage is that substantial savings in cost and time can be achieved by analyzing the cases and only a sample of the controls (as opposed to the entire cohort), particularly when the collection and/or processing of exposure information is very expensive and/or time consuming [6]. While cost is often a major factor in preferring a nested-case control approach over analyzing an entire cohort, there may be advantages even when differences in cost are not significant.

In recent years, large administrative healthcare databases, such as the one from which the example cohort for this study was selected, have become particularly useful in studying outcomes that are very rare because they allow for adequate sample sizes [17-20]. Once a database with all exposure and outcome information is available, analyzing a sample of rather than the entire cohort does not necessarily decrease costs. However, depending on the size of the cohort (as well as the speed of the processor and amount of memory in the computer), it may not be possible to analyze an entire cohort when complex modeling of time-dependent covariates is needed. Such was the case in another study based on a cohort derived from the Quebec provincial healthcare database, where in one analysis the number of covariates was restricted to four and in another analysis only a sub-cohort (i.e. 15529 of 31062 subjects) could be included because the substantial computational resources required were prohibitive [21]. Depending on the rarity of the outcome under study, it may not be possible to analyze the required sample size when performing Cox regression on the whole cohort, whereas it may be possible to do so using a nested case-control approach. While it is recognized that issues of computational resources are overcome with time as computers and software become more efficient, limitations are likely to remain as the size of databases and complexity of time-dependent analyses will also increase.

Both the nested case-control approach described (using conditional logistic regression) and the Cox proportional-hazards model with time-dependent covariates similarly account for the time-dependence of exposure when levels of exposure in subjects vary over time. A different and more complex issue is the possibility that the effect of a given exposure varies over time. This can be addressed by analyzing latency-weighted exposures using either Cox regression or a nested case-control approach, the latter being computationally faster [22]. Alternatively, Cox regression can accommodate changes in the hazard ratio over time with a flexible generalization of the Cox proportional hazards model using a regression spline technique [23-25].

## Conclusions

A nested case-control approach is a useful alternative for analysis of a cohort when time-dependent covariates are used. The expectedly similar risk estimates are obtainable with superior computational efficiency. Particularly when studying the effects of time-dependent exposures on rare outcomes in very large databases, study power can be improved by being able to run complex regression models on a larger number of affected subjects.

## Competing interests

The author(s) declare that they have no competing interests.

## Authors' contributions

All authors participated in the conception and design of the study. VE performed the statistical analysis and drafted the manuscript. All authors contributed to the interpretation of the study and revision of the manuscript. The final manuscript was read and approved by all authors.

## Pre-publication history

The pre-publication history for this paper can be accessed here:


